# Correction to Boeg Thomsen et al. (2021)

**DOI:** 10.1037/dev0001451

**Published:** 2022-09

In the article “Do Complement Clauses Really Support False-Belief Reasoning? A Longitudinal Study With English-Speaking 2- to 3-Year-Olds,” by Ditte Boeg Thomsen, Anna Theakston, Birsu Kandemirci, and Silke Brandt (*Developmental Psychology*, 2021, Vol. 57, No. 8, pp. 1210–1227, https://doi.org/10.1037/dev0001012), the copyright license has been changed to the Creative Commons CC BY Attribution License. The online version of this article has been corrected.

